# Technologies and the Effects On Social Engagement In Long-Term Care Facilities During COVID-19: A Scoping Review

**DOI:** 10.1093/geroni/igab046.3201

**Published:** 2021-12-17

**Authors:** Timothy Wood, Shannon Freeman, Alanna Koopmans

**Affiliations:** 1 University of Northern British Columbia, Canmore, Alberta, Canada; 2 University of Northern British Columbia, Prince George, British Columbia, Canada; 3 UNBC, University of Northern British Columbia, British Columbia, Canada

## Abstract

During the COVID-19 pandemic, the sense of loneliness and social isolation felt by older adults in long-term care facilities has been exacerbated. Although there has been an increase in the number of digital solutions to mitigate social isolation during COVID-19, facilities in northern British Columbia do not have sufficient information regarding the technologies to support social connectedness. To support evidence-based policy decisions, a scoping review was conducted to identify existing virtual technology solutions, apps, and platforms that promote social connectedness among older adults residing in long-term care. A combination of keywords and subject headings were used to identify relevant literature within PubMed, CINAHL EBSCO, PsychINFO EBSCO, Embase OVIDSP, and Web of Science ISI databases. DistillerSR was used to screen and summarize the article selection process. Twenty-three articles were identified for full-text analysis. A variety of technologies are described which can be used to mitigate the impacts of social isolation felt by long-term care residents. However, many of these digital solutions require stable highspeed internet. This remains a challenge for facilities in northern areas as many have limited access to reliable internet. Metrics used to evaluate social engagement in the context of long-term care are also outlined. This research provides the preliminary groundwork necessary to better inform policy decisions about which technologies are available and, of these, which are effective at enhancing social connectedness for older adults in long-term care.

